# Physical Activity and Bidirectional Stage Transitions in Cardiovascular-Kidney-Metabolic Syndrome: A Cohort Study

**DOI:** 10.3390/healthcare14020244

**Published:** 2026-01-19

**Authors:** Chuan Mou, Xinrui Miao, Zhihua Wang

**Affiliations:** 1 Institute of Physical Education, Sichuan University, Chengdu 610065, China; muchuan2012@gmail.com; 2 Institute of Physical Education, Kunsan National University, Gunsan 54150, Republic of Korea; 931905883@office365.kunsan.ac.kr

**Keywords:** physical activity, Cardiovascular-Kidney-Metabolic syndrome, Markov model

## Abstract

Background: Cardiovascular-Kidney-Metabolic (CKM) syndrome involves interconnected cardiovascular, renal, and metabolic conditions. The dose–response relationship between physical activity and bidirectional CKM stage transitions remains unclear. Methods: Using data from the China Health and Retirement Longitudinal Study (CHARLS), cross-sectional analysis pooled 14,310 observations from 10,868 participants. Logistic regression with clustered robust standard errors accounted for intra-individual correlation. Longitudinal analysis (*n* = 3442) employed continuous-time multi-state Markov models with a 5-state structure (Stages 0–4). To evaluate physical activity effects, stages were regrouped into low-risk (Stages 0–2) and high-risk states (Stages 3–4) using a 2 × 2 transition intensity matrix. Physical activity was measured in MET-min/week and categorized into quartiles (Q1–Q4). Results: Compared with Q1, Q2, Q3, and Q4 were associated with 43.1%, 52.5%, and 53.1% lower risk of high-risk CKM stages, respectively. RCS analysis demonstrated nonlinear dose–response relationships between physical activity and CKM stage progression. Subgroup analyses showed more pronounced protective effects in older adults and single individuals. During 4-year follow-up, 31.6% experienced progression and 6.8% showed improvement. Stage 4 acted as a complete absorbing state without any reversal. Transition intensity analysis revealed that transitions between adjacent stages were notably higher than cross-stage transitions. The Q4 physical activity level significantly reduced transitions from low-risk to high-risk states (HR = 0.598, 95% CI: 0.459–0.777) and promoted transitions from high-risk to low-risk states (HR = 2.995, 95% CI: 1.257–7.134). Conclusions: Moderate-to-high physical activity effectively reduces CKM progression risk and promotes improvement, providing evidence for CKM prevention and management.

## 1. Introduction

Complex pathophysiological interactions exist among cardiovascular disease, chronic kidney disease, and metabolic disorders, which mutually influence and accelerate disease progression through shared mechanisms including adipose tissue dysfunction, insulin resistance, systemic inflammation, and oxidative stress [1,2]. In October 2023, the American Heart Association (AHA) formally proposed the Cardiovascular-Kidney-Metabolic (CKM) syndrome framework [3], establishing a five-stage classification system ranging from Stage 0 (no risk factors) to Stage 4 (clinical cardiovascular disease), reflecting the continuum of disease progression. Epidemiological data indicate that approximately 90% of adults possess at least one CKM risk factor, with about one-third having three or more [4,5]. In China, the prevalence of advanced CKM stages (Stages 3–4) reaches 23.6% among adults aged 35 and above [6], reflecting the impact of rapid urbanization, dietary pattern transitions, and sedentary lifestyles on cardiometabolic health in middle-aged and elderly populations [5].

Physical activity has long been recognized as a critical determinant of cardiovascular and metabolic health, with meta-analyses consistently demonstrating its capacity to reduce risks of cardiovascular disease, type 2 diabetes, and all-cause mortality [7,8]. Regarding kidney outcomes, a recent meta-analysis involving 353,975 participants revealed that high levels of physical activity were associated with a 6% reduction in chronic kidney disease risk, with each 10 metabolic equivalent-hours per week (MET-h/week) increase corresponding to a 2% risk reduction [9]. Under the CKM framework, preliminary studies have shown that physical activity provides significant protection across CKM Stages 0–4, with moderate-intensity physical activity exhibiting an L-shaped association with early-stage CKM risk [10]. Additionally, multi-state modeling approaches have been successfully applied to investigate transitions in cardiometabolic conditions such as metabolic syndrome [11] and diabetes progression [12,13].

However, longitudinal evidence specifically examining bidirectional transitions between CKM stages remains notably absent. The dose–response relationship between physical activity intensity and both progression and regression probabilities across CKM stages has not been quantified, particularly in Asian populations. Therefore, this study aimed to address these gaps using data from the China Health and Retirement Longitudinal Study (CHARLS). We employed a complementary dual-analytical approach, combining cross-sectional analysis to identify association patterns between physical activity levels and CKM risk stratification with longitudinal multi-state modeling to elucidate the causal impact of physical activity on bidirectional transitions between CKM stages over time. We hypothesized that greater physical activity would be associated with lower odds of high-risk CKM stages in cross-sectional analysis and slower progression and higher regression probabilities in longitudinal follow-up, with a nonlinear dose–response pattern, providing evidence for CKM prevention and management in middle-aged and elderly populations.

## 2. Materials and Methods

### 2.1. Study Population

Our study was based on data from the CHARLS, a nationally representative large-scale cohort study. CHARLS employed a multi-stage stratified probability sampling method proportional to size and successfully completed the national baseline survey in 2011 [14]. The survey covered 150 counties and 450 villages across 28 provinces nationwide, interviewing 17,708 participants from 10,257 households. These participants, with an average age of 45 years and above, encompassed a diverse population with different genders, lifestyles, behavioral patterns, health conditions, and economic statuses. Follow-up surveys were conducted in 2013 and 2015. The study was approved by the Biomedical Ethics Committee of Peking University (Approval No.: IRB00001052-11015), and all participants provided written informed consent.

This study encompassed two analytical cohorts to evaluate both the cross-sectional association and longitudinal relationship between physical activity and CKM syndrome. For the cross-sectional analysis, physical activity and CKM stage were measured at the same time point. For the longitudinal analysis, baseline (2011) physical activity was used to examine CKM stage transitions from 2011 to 2015. Among participants with missing physical activity data (*n* = 2550), we performed conditional multiple imputation using chained equations (m = 5 imputations) for those with complete key covariates, successfully imputing data for 784 participants. Participants with ≥2 missing covariates (*n* = 1766) were excluded. From the combined sample of both the 2011 and 2015 waves (*n* = 24,914), participants were excluded if they had missing CKM syndrome information (*n* = 12,257), incomplete covariate data (*n* = 23), or inadequate physical activity data after imputation (*n* = 1766). This yielded 10,868 individuals for cross-sectional analysis. For the longitudinal analysis, we further restricted to participants with complete data at both time points, excluding 7426 who participated in only one wave, resulting in a final cohort of 3442 individuals (Figure S1 and Table S1).

### 2.2. Physical Activity Assessment

The CHARLS questionnaire categorized physical activity into three intensity levels: vigorous activities (e.g., running, weightlifting), moderate-intensity activities (e.g., mopping floors, carrying light objects), and light activities (e.g., walking, Tai Chi) [14]. Following the International Physical Activity Questionnaire (IPAQ) guidelines [15,16], we assigned metabolic equivalent (MET) values of 8.0, 4.0, and 3.0 to vigorous, moderate, and light activities, respectively. Participants reported the weekly frequency (days/week) and duration of each activity type. Duration was recorded in five categories (0, 10–29, 30–119, 120–239, ≥240 min), with median values used for calculations (0, 20, 75, 180, 240 min) [17,18]. Total physical activity was calculated by multiplying the MET value of each activity by its duration and frequency, then summing across all activities.

Based on quartile distribution, participants were divided into four groups: Q1 (<1733 MET-min/week, reference), Q2 (1734–5544), Q3 (5545–12,180), and Q4 (>12,180 MET-min/week). Quartile-based categorization was chosen for the primary analysis because it ensures balanced sample sizes across groups, maximizes statistical power, captures dose–response relationships specific to our study population, and avoids arbitrary cutpoint selection that may not reflect the underlying data distribution [19].

### 2.3. Cardiovascular-Kidney-Metabolic (CKM) Syndrome Staging

Our study adopted the CKM syndrome staging criteria published by the American Heart Association in 2023 with population-specific adaptations for Chinese adults (Table S2) [20,21]. CKM syndrome is classified into five stages: Stage 0 represents individuals without CKM risk factors; Stage 1 includes individuals with excess or dysfunctional adiposity; Stage 2 includes individuals with metabolic risk factors and/or moderate-to-severe chronic kidney disease; Stage 3 includes individuals with subclinical cardiovascular disease; and Stage 4 includes individuals with clinical cardiovascular disease. Biochemical indicators for CKM staging were obtained from venous blood samples collected by professional medical staff and analyzed in designated laboratories following standardized protocols.

### 2.4. Covariates

Covariates included sociodemographic characteristics (age, sex, education level, marital status, residence, and retirement status), lifestyle factors (smoking and drinking status), anthropometric measurements, medication use, and disease history [19]. Age was treated as a continuous variable. Education was categorized as primary school and below, middle school, or high school and above. Marital status was classified as married or other (including unmarried, divorced, widowed). Smoking and drinking status were dichotomized as yes/no. Height and weight were measured by trained investigators using standardized instruments, and BMI was calculated and classified as normal/underweight (<24.0 kg/m^2^) or overweight/obese (≥24.0 kg/m^2^) according to Chinese standards. Medication use included antidiabetic, antihypertensive, and lipid-lowering medications. Disease history encompassed diabetes, heart disease (myocardial infarction, coronary heart disease, angina, heart failure), and stroke. Self-reported information was collected through standardized questionnaires administered via face-to-face interviews by trained investigators.

### 2.5. Statistical Analysis

All statistical analyses were performed using R software (version 4.5.0). Continuous variables were expressed as mean (standard deviation) and categorical variables as frequency (percentage). Group comparisons by CKM stages used one-way analysis of variance (ANOVA) for continuous variables and χ^2^ tests for categorical variables. Statistical analyses employed two-sided tests, with *p* < 0.05 considered statistically significant.

#### 2.5.1. Multi-State Markov Model

The primary analysis employed continuous-time multi-state Markov models to assess the impact of physical activity on bidirectional CKM stage transitions, using the msm package in R [22]. We analyzed 3442 participants with complete longitudinal data from 2011 to 2015. The model defined five states corresponding to CKM Stages 0–4, with Stages 0–3 as transient states allowing transitions to any other state, and Stage 4 as an absorbing state. The absorbing state specification for Stage 4 was based on both clinical rationale and empirical observation: clinically, Stage 4 represents established cardiovascular disease (myocardial infarction, heart failure, stroke) which is generally irreversible; empirically, 100% of Stage 4 participants in our cohort remained in this stage with no reversals observed during follow-up, consistent with prior multi-state modeling in cardiovascular populations [2]. The model allowed all theoretically possible transition paths, including adjacent-stage transitions, cross-stage transitions, and bidirectional transitions. Transition intensity parameters were estimated using quasi-Newton optimization and maximum likelihood estimation, with transition probability matrices calculated through matrix exponential functions.

To evaluate the effect of physical activity on clinically meaningful transitions, CKM stages were regrouped into low-risk (Stages 0–2) and high-risk (Stages 3–4) states using a 2 × 2 transition intensity matrix. This dichotomization aligns with clinical decision-making thresholds where Stages 3–4 typically warrant more intensive intervention and enhances model stability by reducing parameter dimensionality. Baseline (2011) physical activity quartiles were incorporated as time-independent covariates, calculating hazard ratios for deterioration transitions (low-risk → high-risk) and improvement transitions (high-risk → low-risk).

#### 2.5.2. Secondary Analyses

Cross-sectional analysis. We used multivariable logistic regression models with clustered robust standard errors (clustering by individual participant ID) to account for correlation between repeated measurements from individuals contributing data at both 2011 and 2015. Three progressively adjusted models were constructed: Model 1 (unadjusted); Model 2 (adjusted for age, sex, and BMI); and Model 3 (further adjusted for marital status, retirement status, residence, education level, smoking status, drinking status, medication use, and disease history). Results were expressed as odds ratios (OR) with 95% confidence intervals, with Q1 as reference.

Dose–response analysis. To explore nonlinear relationships between total MET-min/week and CKM stage progression, restricted cubic spline (RCS) analysis was employed within Model 3. Three knots were set at the 10th, 50th, and 90th percentiles, with the 10th percentile as reference. RCS analyses were performed for four CKM stage comparisons (Stages 0–1 vs. 2–4, Stages 0–2 vs. 3–4, Stages 0 vs. 1–4, Stages 0–3 vs. 4) to evaluate protective effects across the CKM spectrum.

Subgroup analysis. Stratified analyses based on Model 3 explored heterogeneity of physical activity effects across age, sex, BMI, marital status, residence, smoking, and drinking status. Interaction tests assessed effect modification. These analyses were exploratory without adjustment for multiple comparisons.

### 2.6. Sensitivity Analyses

Multiple sensitivity analyses were conducted to test result robustness. First, we added CESD-10 depression scores, lung disease, and C-reactive protein as additional covariates. Second, we reassessed associations using IPAQ classification standards (low: <600, moderate: 600–3000, high: >3000 MET-min/week). Third, total physical activity was analyzed as a continuous variable (per thousand MET-min/week). Fourth, we used alternative CKM groupings (Stages 2–4 vs. 0–1) in both logistic regression and Markov models. Fifth, a nested case–control study matched high-risk cases (Stages 3–4) to low-risk controls (Stages 0–2) using propensity scores (1:3 ratio, caliper 0.1) based on demographic and clinical characteristics.

## 3. Results

### 3.1. Baseline Characteristics

This study comprised a cross-sectional analysis cohort (10,868 participants, 21,736 observations) and a longitudinal multi-state model analysis cohort (3442 participants, 6884 observations), both derived from two survey waves in 2011 and 2015. Baseline characteristics were similar between the two cohorts. The cross-sectional cohort had a mean age of 58.63 ± 8.72 years with 53.7% females. The longitudinal cohort had a mean age of 59.45 ± 8.12 years with 55.2% females. Stage 2 was the predominant CKM stage distribution in both cohorts. All baseline characteristics showed statistically significant differences across CKM stages (*p* < 0.001). Total metabolic equivalents and the proportion of participants with high physical activity levels showed progressive declines from Stage 0 to Stage 4, while the proportion with low physical activity levels increased significantly in advanced CKM stages (Table 1 and Table S3).

### 3.2. Association Between Physical Activity and High-Risk CKM Stages

**Figure 1:**
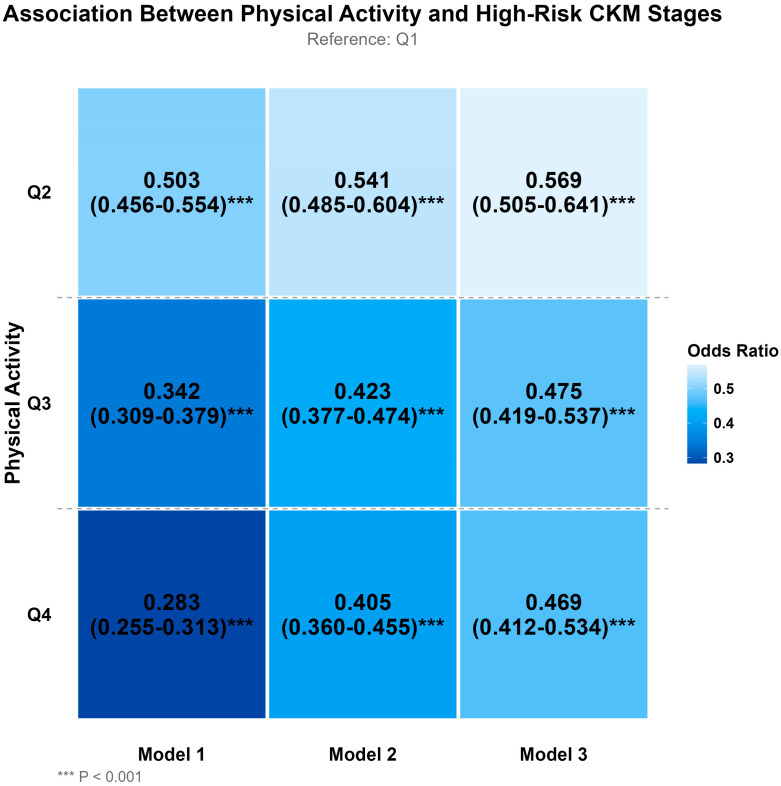
Association between physical activity and high-risk Cardiovascular-Kidney-Metabolic (CKM) Stages. Notes: ORs and 95% CI for the association between physical activity and high-risk CKM stages (Stages 3–4 vs. Stages 0–2). Reference group: Q1 (lowest physical activity level, <1733 MET-min/week). All associations are statistically significant (*** *p* < 0.001). Model 1: No adjustment for any covariates (crude model). Model 2: Adjusted for sociodemographic and anthropometric factors including age, gender, and BMI. Model 3: Adjusted for age, gender, BMI, sociodemographic factors (marital status, retirement status, residence, education level), lifestyle factors (smoking and drinking status), medication use (antidiabetic medication, antihypertensive medication, lipid-lowering medication), and disease history (stroke, diabetes, heart disease). Physical activity categories: Q1: <1733 MET-min/week (reference); Q2: 1734–5544 MET-min/week; Q3: 5545–12,180 MET-min/week; Q4: >12,180 MET-min/week.

Figure 1 illustrates the association between physical activity levels and high-risk CKM stages (Stages 3–4 vs. Stages 0–2). Higher levels of physical activity showed significant inverse associations with high-risk CKM stages across all models, presenting a clear dose–response relationship (all *p* < 0.001). After full adjustment in Model 3, the risks of high-risk CKM stages in Q2, Q3, and Q4 groups were reduced by 43.1% (OR = 0.569, 95% CI: 0.505–0.641, *p* < 0.001), 52.5% (OR = 0.475, 95% CI: 0.419–0.537, *p* < 0.001), and 53.1% (OR = 0.469, 95% CI: 0.412–0.534, *p* < 0.001), respectively, compared with Q1.

**Figure 2:**
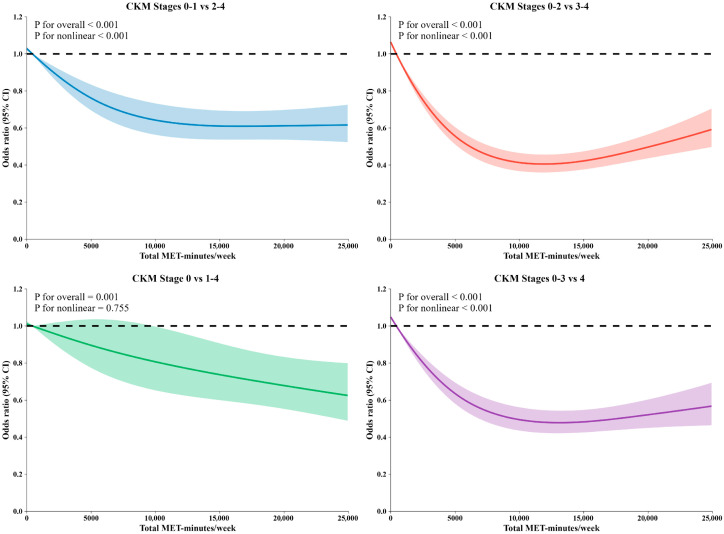
Dose–response relationships between physical activity and Cardiovascular-Kidney-Metabolic (CKM) stage progression. Notes: Model adjusted for age, gender, BMI, sociodemographic factors (marital status, retirement status, residence, education level), lifestyle factors (smoking and drinking status), medication use (antidiabetic medication, antihypertensive medication, lipid-lowering medication), and disease history (stroke, diabetes, heart disease).

RCS analysis revealed distinct dose–response patterns across CKM stage comparisons (Figure 2). For CKM Stages 0–1 vs. 2–4, Stages 0–2 vs. 3–4, and Stages 0–3 vs. 4, all showed significant overall associations and nonlinear relationships (P overall < 0.001, P nonlinear < 0.001). In contrast, Stages 0 vs. 1–4 showed a linear protective effect (P nonlinear = 0.755).

**Figure 3:**
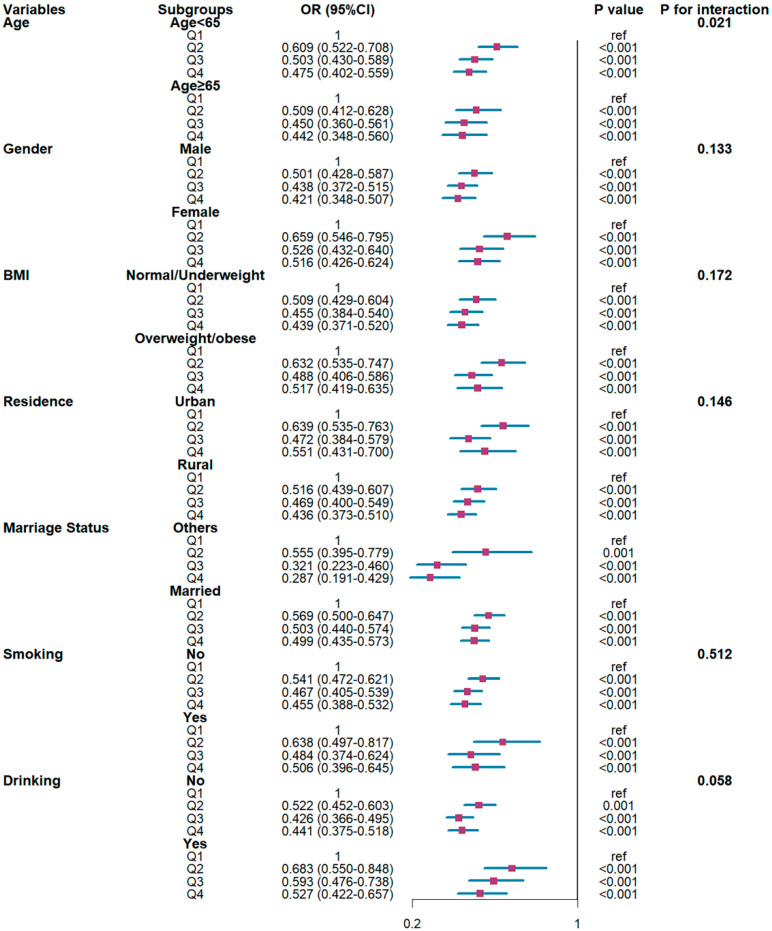
Stratified analysis of the association between physical activity and high-risk Cardiovascular-Kidney-Metabolic (CKM) stages.

Subgroup analyses identified two significant effect modifiers (Figure 3). Age significantly modified the association (P interaction = 0.021), with stronger protection in adults ≥ 65 years. Marital status also showed significant interaction (P interaction = 0.020). Single individuals derived greater benefit from high physical activity compared to married individuals. No significant interactions were observed for sex, BMI, smoking, drinking, or residence (P interaction > 0.05).

### 3.3. Physical Activity and CKM Stage Transition Patterns

During 4-year follow-up, 61.6% of participants remained stable, 31.6% experienced progression, and 6.8% showed improvement. At follow-up, CKM stage distribution was as follows: Stage 0 (2.4%), Stage 1 (13.9%), Stage 2 (47.9%), Stage 3 (14.2%), and Stage 4 (21.4%).

**Figure 4:**
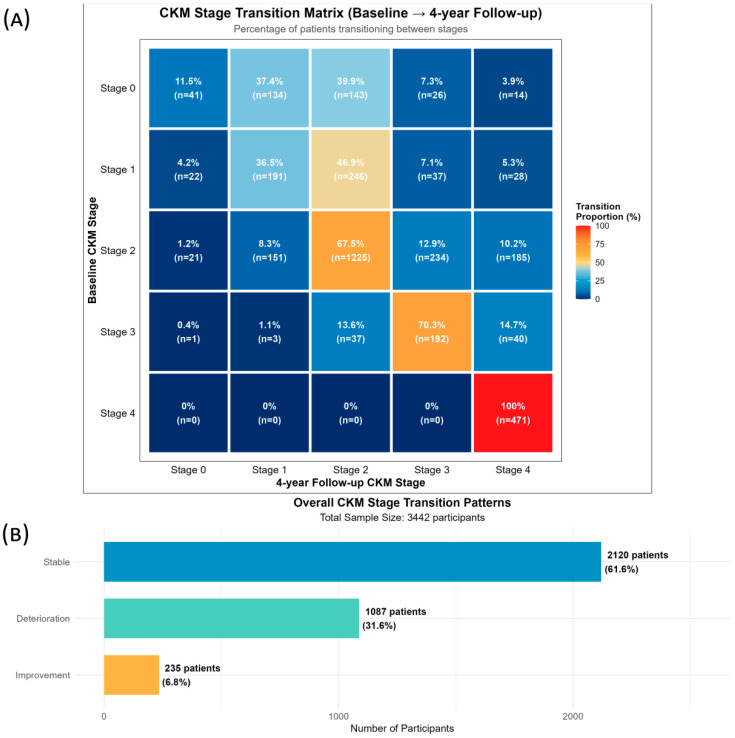
Cardiovascular-Kidney-Metabolic (CKM) stage transition matrix and overall transition patterns during 4-year follow-up. (**A**) Percentage and number of participants transitioning between CKM stages from baseline (2011) to 4-year follow-up (2015). Rows represent baseline CKM stages, and columns represent follow-up stages. Colors indicate transition proportions, with diagonal cells (same stage) showing stability, off-diagonal cells above the diagonal showing disease progression, and cells below the diagonal showing disease improvement. (**B**) Overall CKM stage transition patterns categorized into three types.

Key transition patterns are summarized in Figure 4 and Table S4. Stage 2 exhibited the highest stability (67.5%). Stage 4 was completely absorbing with 100% stability and no reversals observed. Transition intensity analysis (Table 2) confirmed that adjacent-stage transitions predominated over cross-stage transitions: Stages 0 → 1 intensity was 24.8 times higher than Stages 0 → 3. Reversal became increasingly difficult at higher stages, with Stage 3 showing the lowest reversal intensities.

**Figure 5:**
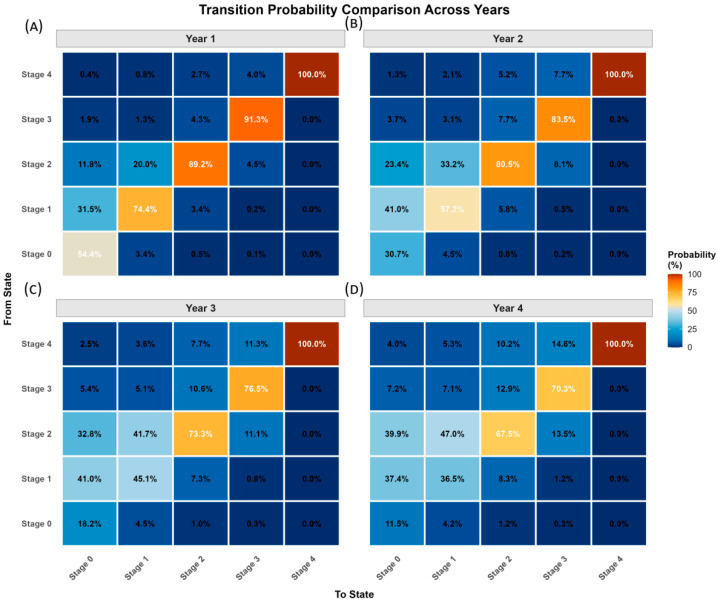
Transition probabilities of Cardiovascular-Kidney-Metabolic (CKM) stages across different time intervals. Transition probabilities between CKM stages at 1-year (**A**), 2-year (**B**), 3-year (**C**), and 4-year (**D**) observation intervals based on multi-state Markov models. Colors represent transition probabilities, with darker blue indicating lower probability and orange/red indicating higher probability. The diagonal cells represent the probability of remaining in the same stage. Numbers shown are percentages of participants transitioning from baseline CKM stage (rows) to follow-up stage (columns). CKM, Cardiovascular-Kidney-Metabolic syndrome.

Transition probability analysis (Figure 5) revealed time-dependent patterns. Stage 0 stability decreased from 54.4% (1-year) to 11.5% (4-year). Stage 1 showed a critical window for intervention: reversal to Stage 0 dropped from 31.5% to 4.2% over 4 years, while progression to Stage 2 increased from 3.4% to 46.9%. Stage 2 maintained the highest long-term stability (67.5% at 4 years).

The effects of physical activity on hazard ratios (HRs) for transitions between low-risk states (Stages 0–2) and high-risk states (Stages 3–4) are shown in Table 3. Compared with the Q1 reference group, for deterioration transitions (low-risk → high-risk), the Q3 and Q4 groups significantly reduced deterioration risk by 22.2% and 40.2%, respectively, while Q2 showed no statistical significance. For improvement transitions (high-risk → low-risk), only the Q4 group significantly promoted disease improvement, increasing the likelihood of improvement by nearly 3-fold (HR = 2.995, 95% CI: 1.257–7.134).

### 3.4. Sensitivity Analysis

Multiple sensitivity analyses confirmed robustness of findings (Tables S5–S10). Results remained consistent using propensity score matching, IPAQ classification criteria, continuous physical activity variables, and alternative CKM groupings. The nested case–control study showed consistent protective effects across Q2–Q4 groups (*p* < 0.001). When using broader grouping criteria (Stages 2–4 vs. 0–1), only Q4 showed significant protection, suggesting higher activity thresholds for preventing earlier-stage progression.

## 4. Discussion

This study demonstrated a clear dose–response relationship between physical activity and CKM syndrome risk and represents the first application of multi-state Markov models to characterize bidirectional CKM stage transitions. Our findings indicate that moderate-to-high physical activity not only reduces progression risk but also promotes regression from high-risk to low-risk states.

### 4.1. Comparison with Previous Literature

Our cross-sectional findings align with and extend previous research. A large-scale study demonstrated that achieving recommended physical activity levels resulted in a 29% reduction in cardiovascular disease mortality [23]. A Chinese cohort study reported that high-intensity physical activity reduced CKM progression risk by 27.5% [24]. Our observed risk reductions of 43–53% in high-risk CKM stages are comparable to these estimates, though direct comparison is limited by differences in outcome definitions and population characteristics. The nonlinear dose–response pattern we identified is consistent with current World Health Organization (WHO) and AHA guidelines [25,26], which recommend 150–300 min/week of moderate-intensity activity. The linear relationship observed for Stage 0 vs. Stages 1–4, compared with nonlinear patterns for more advanced stage comparisons, suggests that physical activity’s protective mechanisms may differ across the CKM spectrum.

Our longitudinal findings address a critical gap in the literature. Since the AHA proposed the CKM staging system in 2023, evidence on dynamic disease transitions has been lacking [2]. The predominance of adjacent-stage over cross-stage transitions is consistent with multi-state models in US Medicare populations [27], supporting the concept of CKM syndrome as a progressive continuum. The observed regression potential in Stages 1–3 parallels diabetes remission research, where intensive lifestyle intervention achieved 7% remission rates—six times higher than controls [28]. Similarly, the US Diabetes Prevention Program found that meeting physical activity targets reduced diabetes incidence by 44% independent of weight loss [29].

The stronger protective effects in elderly and unmarried populations are consistent with previous findings. Meta-analyses have shown 19–30% reductions in all-cause mortality among elderly individuals meeting activity recommendations [30]. The effect modification by marital status may reflect differences in social support structures and health behaviors [31].

### 4.2. Interpretation of Findings

The observed protective effects can be explained by physical activity’s multi-system biological impacts, including improved insulin sensitivity through Glucose transporter type 4 (GLUT4) translocation [32], enhanced cardiovascular function via angiogenesis and endothelial improvement [33], reduced systemic inflammation through visceral fat reduction [34], and improved renal hemodynamics [35]. These synergistic mechanisms collectively explain the robust cross-organ protection against CKM syndrome.

The threshold effect observed in longitudinal analysis—where only Q4 significantly promoted improvement transitions—suggests that moderate physical activity may be sufficient to prevent disease progression, whereas disease regression may require more substantial, sustained activity levels. This finding has important implications for clinical recommendations, indicating that prevention and reversal may require different activity thresholds.

### 4.3. Clinical and Public Health Implications

Our findings have several implications for clinical practice and public health policy. Routine CKM evaluation should incorporate physical activity assessment as a modifiable risk factor, and clinicians should encourage inactive individuals to achieve at least 150 min/week of moderate-intensity activity, as this threshold provides substantial protection with diminishing returns beyond 250–300 min/week. Given the stronger protective effects observed in elderly and unmarried individuals, these populations may benefit most from targeted physical activity promotion programs. Perhaps most importantly, the demonstrated reversibility of Stages 1–3, contrasted with the irreversible nature of Stage 4, underscores the critical importance of early screening and intervention before progression to advanced disease stages.

### 4.4. Strengths and Limitations

Strengths of this study include the large-scale nationally representative cohort, the novel application of multi-state Markov models to CKM research, and the complementary cross-sectional and longitudinal analyses that strengthen causal inference.

Several limitations should be acknowledged. First, self-reported physical activity may introduce recall and social desirability bias, potentially attenuating observed associations; objective accelerometer-based measurement would strengthen future studies. Second, odds ratios may overestimate associations given the high prevalence of high-risk CKM stages. Third, baseline-only physical activity assessment cannot capture activity changes during follow-up. Fourth, residual confounding (e.g., dietary patterns) cannot be excluded despite multivariable adjustment. Fifth, findings from this middle-aged and elderly Chinese population may not generalize to other populations. Sixth, with only two time points, refined transition dynamics could not be captured, and the observed stage “regression” may partially reflect measurement variability near classification thresholds or regression to the mean rather than sustained disease reversal [36]. Seventh, subgroup analyses were exploratory without multiple comparison correction, and these findings require confirmation [37]. Finally, despite the longitudinal design, reverse causation cannot be excluded without randomized controlled trials.

Future research should incorporate objective physical activity measurement, more frequent follow-up assessments, and diverse populations to validate and extend these findings. Randomized controlled trials examining physical activity interventions across CKM stages would provide definitive causal evidence.

## 5. Conclusions

This study provides the first systematic evaluation of associations between physical activity and bidirectional transitions in CKM syndrome stages. Our findings demonstrate that moderate-to-high levels of physical activity are associated with lower risk of CKM stage progression and higher probability of disease improvement, supporting the partial reversibility of early-stage CKM syndrome. These findings suggest potential benefits of incorporating physical activity promotion into CKM prevention and management strategies, particularly for middle-aged and elderly populations.

## Figures and Tables

**Table 1:** Baseline demographic and clinical characteristics of participants by CKM stage (cross-sectional analysis cohort, *n* = 10,868).

Variables	CKM Stage						*p*-Value
	Overall	0	1	2	3	4	
No. of participants	10,868	858	1616	5389	1241	1764	
Age, year	58.63 (8.72)	54.61 (7.69)	54.94 (7.16)	56.99 (7.41)	69.58 (6.76)	61.27 (8.53)	<0.001
Age group							<0.001
<55	8047 (74.0)	759 (88.5)	1449 (89.7)	4451 (82.6)	261 (21.0)	1127 (63.9)	
≥65	2821 (26.0)	99 (11.5)	167 (10.3)	938 (17.4)	980 (79.0)	637 (36.1)	
Gender							<0.001
Female	5836 (53.7)	369 (43.0)	777 (48.1)	3229 (59.9)	431 (34.7)	1030 (58.4)	
Male	5032 (46.3)	489 (57.0)	839 (51.9)	2160 (40.1)	810 (65.3)	734 (41.6)	
BMI, kg/m^2^	24.09 (3.34)	21.54 (1.56)	23.32 (2.96)	24.52 (3.34)	23.79 (3.25)	24.93 (3.59)	<0.001
BMI group							<0.001
Normal/Underweight	5205 (47.9)	742 (86.5)	905 (56.0)	2263 (42.0)	629 (50.7)	666 (37.8)	
Overweight/obese	5663 (52.1)	116 (13.5)	711 (44.0)	3126 (58.0)	612 (49.3)	1098 (62.2)	
Residence							<0.001
Urban	4063 (37.4)	274 (31.9)	522 (32.3)	2035 (37.8)	464 (37.4)	768 (43.5)	
Rural	6805 (62.6)	584 (68.1)	1094 (67.7)	3354 (62.2)	777 (62.6)	996 (56.5)	
Retirement status							<0.001
No	9583 (88.2)	791 (92.2)	1497 (92.6)	4878 (90.5)	1009 (81.3)	1408 (79.8)	
Yes	1285 (11.8)	67 (7.8)	119 (7.4)	511 (9.5)	232 (18.7)	356 (20.2)	
Marriage Status							<0.001
Others	1182 (10.9)	56 (6.5)	110 (6.8)	491 (9.1)	282 (22.7)	243 (13.8)	
Married	9686 (89.1)	802 (93.5)	1506 (93.2)	4898 (90.9)	959 (77.3)	1521 (86.2)	
Current Smoking							<0.001
No	6541 (60.2)	449 (52.3)	946 (58.5)	3596 (66.7)	468 (37.7)	1082 (61.3)	
Yes	4327 (39.8)	409 (47.7)	670 (41.5)	1793 (33.3)	773 (62.3)	682 (38.7)	
Current Drinking							<0.001
No	6293 (57.9)	479 (55.8)	895 (55.4)	3245 (60.2)	600 (48.3)	1074 (60.9)	
Yes	4575 (42.1)	379 (44.2)	721 (44.6)	2144 (39.8)	641 (51.7)	690 (39.1)	
Physical Activity							<0.001
Total MET	7556.82 (6924.91)	10,060.75 (7379.80)	9176.29 (7072.52)	7793.69 (6818.22)	5703.28 (6528.30)	5435.71 (6220.51)	<0.001
Q1 (<1733)	3050 (28.1)	165 (19.2)	323 (20.0)	1242 (23.0)	553 (44.6)	767 (43.5)	
Q2 (1734–5544)	2432 (22.4)	148 (17.2)	320 (19.8)	1284 (23.8)	260 (21.0)	420 (23.8)	
Q3 (5545–12,180)	2677 (24.6)	170 (19.8)	361 (22.3)	1615 (30.0)	213 (17.2)	318 (18.0)	
Q4 (>12,180)	2709 (24.9)	375 (43.7)	612 (37.9)	1248 (23.2)	215 (17.3)	259 (14.7)	

Abbreviations: CKM, Cardiovascular-Kidney-Metabolic; BMI, body mass index; MET, metabolic equivalent of task. Note: Data are presented as mean (standard deviation) for continuous variables and *n* (%) for categorical variables. Others in Marriage Status include widowed, divorced, separated, and never married.

**Table 2:** Transition intensities for deterioration and recovery between CKM Stages.

Transition Type	From		To	Transition Intensity (95% CI)
Deteriorate transitions				
	Stage 0	→	Stage 1	0.496 (0.366–0.674)
			Stage 2	0.108 (0.046–0.252)
			Stage 3	0.021 (0.005–0.085)
			Stage 4	0.002 (0.001–56.843)
	Stage 1	→	Stage 2	0.244 (0.207–0.288)
			Stage 3	0.010 (0.003–0.041)
			Stage 4	0.007 (0.002–0.026)
	Stage 2	→	Stage 3	0.048 (0.041–0.055)
			Stage 4	0.028 (0.023–0.033)
	Stage 3	→	Stage 4	0.042 (0.030–0.058)
Recovery transitions				
	Stage 1	→	Stage 0	0.054 (0.03–0.097)
	Stage 2			0.006 (0.003–0.015)
	Stage 3			0.002 (0.001–0.044)
	Stage 2	→	Stage 1	0.042 (0.033–0.052)
	Stage 3	→	Stage 0	0.002 (0.001–0.044)
			Stage 1	0.001 (0.001–29.628)
			Stage 2	0.050 (0.036, 0.070)
	Stage 4	→	Stage 2	0.001 (0.001–1.173)
			Stage 3	0.001 (0.001–1.256)

Notes: Transition intensities represent the instantaneous hazard of transitioning from one CKM stage to another. Values in parentheses represent 95% confidence intervals. CKM, Cardiovascular-Kidney-Metabolic syndrome; CI, confidence interval.

**Table 3:** Hazard ratios for bidirectional transitions between low-risk states (Stages 0–2) and high-risk states (Stages 3–4) by physical activity quartiles.

Hazard Ratio (95% CI)		
Physical activity	Transition Type	
	Deteriorate transitions	Recovery transitions
	State 1 → State 2	State 2 → State 1
Q1	Ref	Ref
Q2	1.012 (0.800–1.280)	2.198 (0.922–5.243)
Q3	0.778 (0.611–0.990)	1.681 (0.646–4.370)
Q4	0.598 (0.459–0.777)	2.995 (1.257–7.134)

Notes: Hazard ratios (HRs) represent the instantaneous hazard of transitioning from one CKM state to another. Values in parentheses represent 95% confidence intervals. State 1 represents low-to-moderate risk CKM stages (Stages 0–2); State 2 represents high-risk CKM stages (Stages 3–4). CKM, Cardiovascular-Kidney-Metabolic; CI confidence interval.

## Data Availability

The dataset analyzed in this study can be accessed through the China Health and Retirement Longitudinal Study repository at https://charls.pku.edu.cn/ (accessed on 15 January 2026).

## Supplementary Materials

The following supporting information can be downloaded at: https://www.mdpi.com/article/10.3390/healthcare14020244/s1, Table S1: Summary of study design and analytical cohorts. Table S2. CKM syndrome staging criteria with Chinese population adaptations. Table S3. Baseline Demographic and Clinical Characteristics of Participants by CKM Stage (Longitudinal Multi-State Model Analysis Cohort, *n* = 3442). Table S4. Exact counts of CKM stage transitions from baseline to 4-year follow-up. Table S4. Exact counts of CKM stage transitions from baseline to 4-year follow-up. Table S6. Association between physical activity levels (based on IPAQ criteria) and high-risk CKM Stages. Table S7. Association between physical activity (per 1000 MET-min/week) and high-risk CKM Stages. Table S8. Association between physical activity and high-risk CKM Stages (Stages 2–4 vs. Stages 0–1). Table S9. Association between physical activity and high-risk CKM Stages: Results from propensity score matching analysis. Table S10. Hazard ratios for bidirectional CKM Stage transitions by physical activity quartiles. Figure S1. Flow chart of participant selection for cross-sectional and longitudinal analyses.

## Abbreviations

CKM	Cardiovascular-Kidney-Metabolic
AHA	American Heart Association
CHARLS	China Health and Retirement Longitudinal Study
IPAQ	International Physical Activity Questionnaire
BMI	Body mass index
ANOVA	Analysis of variance
ORs	Odds ratios
CIs	Confidence intervals
RCS	Restricted cubic spline
CKD	Chronic kidney disease
CVD	Cardiovascular disease
CESD-10	Center for Epidemiologic Studies Depression Scale 10-item
GLUT4	Glucose transporter type 4
HR	Hazard ratio
MET	Metabolic equivalent
WHO	World Health Organization
