# Supplementary material for: Physical Activity and Bidirectional Stage Transitions in Cardiovascular-Kidney-Metabolic Syndrome: A Cohort Study
Source: Healthcare (Basel). 2026 Jan 19;14(2):244. doi: 10.3390/healthcare14020244 (PMC12841378; doi:10.3390/healthcare14020244)
Supplement: Supplementary file 1 [file healthcare-14-00244-s001.zip › healthcare-4069647-supplementary.pdf]

Supplementary Material

1.Supplementary Tables

Table S1. Summary of study design and analytical cohorts.

Analysis Type	Sample Size	Data Structure	Primary Outcome	Statistical Method
Cross-sectional	14310 observations from 10868 individuals	Pooled 2011 and 2015 data	High-risk CKM stages (Stage 3-4)	Logistic regression with clustered robust SE
Longitudinal	3442 individuals	2011-2015 follow-up	CKM stage transitions	Multi-state Markov model

Table S2. CKM syndrome staging criteria with Chinese population adaptations.

Stage	Definition	Chinese-Specific Criteria
Stage 0	No CKM risk factors	Normal BMI, waist circumference, blood glucose, blood pressure, lipids, and kidney function
Stage 1	Excess/dysfunctional adiposity	BMI ≥24 kg/m²; Waist circumference: men ≥90 cm, women ≥85 cm; or prediabetes
Stage 2	Metabolic risk factors and/or CKD	Metabolic syndrome, diabetes, hypertension, dyslipidemia, or CKD (eGFR <60 mL/min/1.73 m² or UACR ≥30 mg/g)
Stage 3	Subclinical CVD	10-year CVD risk ≥20% (PREVENT model) or high-risk CKD, based on Stage 1 or 2
Stage 4	Clinical CVD	Physician-diagnosed coronary heart disease, myocardial infarction, heart failure, or stroke

Table S3. Baseline Demographic and Clinical Characteristics of Participants by CKM Stage (Longitudinal Multi-State Model Analysis Cohort, n=3442)

Variables	CKM stage						*P*-value
	Overall	0	1	2	3	4	
**Observations**	6884	443	1003	3467	762	1209	
**Age, year**	59.45(8.12)	55.57(7.42)	56.07(7.11)	58.05(7.00)	68.79(6.53)	61.81(8.08)	<0.001
**Age group**							<0.001
<55	4989(72.5)	388(87.6)	877(87.4)	2791(80.5)	172(22.6)	761(62.9)	
≥65	1895(27.5)	55(12.4)	126(12.6)	676(19.5)	590(77.4)	448(37.1)	
**Gender**							<0.001
Female	3800(55.2)	184(41.5)	490(48.9)	2155(62.2)	262(34.4)	709(58.6)	
Male	3084(44.8)	259(58.5)	513(51.1)	1312(37.8)	500(65.6)	500(41.4)	
**BMI, kg/m²**	24.24(3.27)	21.68(1.55)	23.34(2.85)	24.52(3.27)	24.28(3.15)	25.12(3.50)	<0.001
**BMI group**							<0.001
Normal/Underweight	3115(45.2)	370(83.5)	560(55.8)	1440(41.5)	326(42.8)	419(34.7)	
Overweight/obese	3769(54.8)	73(16.5)	443(44.2)	2027(58.5)	436(57.2)	790(65.3)	
**Residence**							<0.001
Urban	2410(35.0)	126(28.4)	324(32.3)	1228(35.4)	268(35.2)	464(38.4)	

Rural	4474(65.0)	317(71.6)	679(67.7)	2239(64.6)	494(64.8)	745(61.6)	
**Retirement status**							<0.001
No	Not_retired	6159(89.5)	409(92.3)	931(92.8)	3156(91.0)	644(84.5)	
Yes	Retired	725(10.5)	34(7.7)	72(7.2)	311(9.0)	118(15.5)	
**Marriage Status**							<0.001
Others	717(10.4)	25(5.6)	77(7.7)	301(8.7)	148(19.4)	166(13.7)	
Married	6167(89.6)	418(94.4)	926(92.3)	3166(91.3)	614(80.6)	1043(86.3)	
Current **Smoking**							<0.001
No	4211(61.2)	237(53.5)	606(60.4)	2383(68.7)	254(33.3)	731(60.5)	
Yes	2673(38.8)	206(46.5)	397(39.6)	1084(31.3)	508(66.7)	478(39.5)	
Current **Drinking**							<0.001
No	4010(58.3)	242(54.6)	569(56.7)	2113(60.9)	348(45.7)	738(61.0)	
Yes	2874(41.7)	201(45.4)	434(43.3)	1354(39.1)	414(54.3)	471(39.0)	
**Physical Activity**							<0.001
Total MET	7617.73(6878.75)	10121.16(7312.81)	9446.71(7081.56)	7816.40(6781.80)	6096.81(6581.54)	5571.95(6193.12)	<0.001
Q1 (< 1733)	1848(26.8)	86(19.4)	185(18.4)	782(22.6)	288(37.8)	507(41.9)	
Q2 (1734-5544)	1617(23.5)	76(17.2)	196(19.5)	868(25.0)	188(24.7)	289(23.9)	
Q3 (5545-12180)	1699(24.7)	91(20.5)	230(22.9)	1005(29.0)	153(20.1)	220(18.2)	
Q4 (> 12180)	1720(25.0)	190(42.9)	392(39.1)	812(23.4)	133(17.5)	193(16.0)	

Abbreviations: CKM, cardiovascular-kidney-metabolic; BMI, body mass index; MET, metabolic equivalent of task.

Note: Data are presented as mean (standard deviation) for continuous variables and n (%) for categorical variables. Others in Marriage Status includes widowed, divorced, separated, and never married.

Table S4. Exact counts of CKM stage transitions from baseline to 4-year follow-up.

Baseline Stage	Stage 0	Stage 1	Stage 2	Stage 3	Stage 4	Total
Stage 0	41	134	143	26	14	358
Stage 1	22	191	246	37	28	524
Stage 2	21	151	1225	234	185	1816
Stage 3	1	3	37	192	40	273
Stage 4	0	0	0	0	471	471

Note: Values represent the number of participants transitioning from baseline CKM stage (rows) to 4-year follow-up stage (columns). Diagonal cells (highlighted in light blue) indicate participants who remained in the same stage. Total column shows baseline sample size for each stage. Total sample size: n = 3442 participants.

Table S5. Association between physical activity and high-risk CKM Stages after adjusting extra covariates.

Physical activity	+ CESD 10		+ Lung disease		+ C-reactive protein	
	OR (95% CI)	p-value	OR (95% CI)	p-value	OR (95% CI)	p-value
Q1	1	Ref	1	Ref	1	Ref
Q2	0.578 (0.513, 0.652)	<0.001	0.572 (0.507, 0.644)	<0.001	0.570 (0.505, 0.642)	<0.001
Q3	0.483 (0.426, 0.547)	<0.001	0.475 (0.419, 0.538)	<0.001	0.475 (0.419, 0.538)	<0.001
Q4	0.471 (0.413, 0.537)	<0.001	0.470 (0.412, 0.535)	<0.001	0.470 (0.413, 0.535)	<0.001

Table S6. Association between physical activity levels (based on IPAQ criteria) and high-risk CKM Stages.

Physical activity	OR (95% CI)	p-value
Low （<600）	1	Ref
Moderate（600-3000）	0.891 (0.773, 1.028)	0.114
High（>3000）	0.496 (0.439, 0.560)	<0.001

Table S7. Association between physical activity (per 1000 MET-min/week) and high-risk CKM Stages.

Physical activity	Model 1		Model 2		Model 3	
	OR (95% CI)	*p*-value	OR (95% CI)	*p*-value	OR (95% CI)	*p*-value
Per 1000-unit increase	0.937 (0.931, 0.943)	<0.001	0.958 (0.951, 0.964)	<0.001	0.966 (0.959, 0.973)	0.002

Model 1, no adjustment for any covariates.

Model 2, adjusted for age, gender, BMI.

Model 3, adjusted for age, gender, BMI, sociodemographic factors (marital status, retirement status, residence, education level), lifestyle factors (smoking and drinking status), medication use (antidiabetic medication, antihypertensive medication, lipid-lowering medication), and disease history (stroke, diabetes, heart disease).

Table S8. Association between physical activity and high-risk CKM Stages (Stage 2-4 vs Stage 0-1).

Physical activity	OR (95% CI)	p-value
Q1	1	Ref
Q2	0.984 (0.854, 1.135)	0.827
Q3	1.085 (0.943, 1.249)	0.255
Q4	0.567 (0.497, 0.646)	<0.001

Table S9. Association between physical activity and high-risk CKM Stages: Results from propensity score matching analysis.

Physical activity	OR (95% CI)	p-value
Q1	1	Ref
Q2	0.503 (0.457, 0.554)	<0.001
Q3	0.378 (0.340, 0.420)	<0.001
Q4	0.406 (0.362, 0.454)	<0.001

Table S10. Hazard ratios for bidirectional CKM Stage transitions by physical activity quartiles.

Hazard Ratio (95% CI)		
	Transition Type	
**Physical activity**	Deteriorate transitions	Recovery transitions
	State 1 → State 2	State 2 → State 1
Q1	Ref	Ref
Q2	1.101 (0.813-1.490)	1.449 (0.892-2.352)
Q3	1.048 (0.789-1.393)	1.508 (0.933-2.438)
Q4	1.164 (0.885-1.530)	2.579 (1.624-4.095)

Notes: Hazard ratios (HRs) represent the instantaneous hazard of transitioning from one CKM state to another. Values in parentheses represent 95% confidence intervals. State 1 represents low-to-moderate risk CKM stages (Stage 0-1); State 2 represents high-risk CKM stages (Stage 2-4). *CKM* cardiovascular-kidney-metabolic; *CI* confidence interval.

2. Supplementary Figures

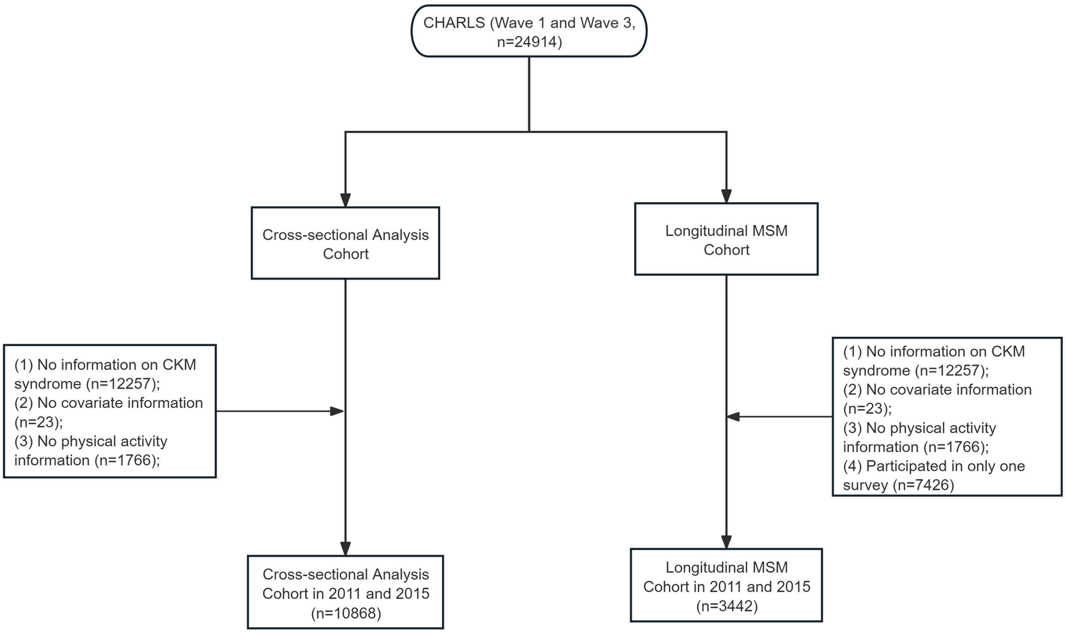

Figure S1. Flow chart of participant selection for cross-sectional and longitudinal analyses.
